# In the Arab Bedroom: The Sex Life of Arabs

**Published:** 2016-12

**Authors:** P Dupont

**Affiliations:** Hoogstraat 134, Genk, Belgium

**Keywords:** Arabs, sex life, sexual intimacy

## Abstract

The sex life of Arabs is terra incognita for scientists and policy makers. Shereen El Feki’s book “Sex and the Citadel” is the first serious attempt to chart sexual intimacy in the rapidly changing Arab world.

“I swear by God, there is a genuine need for knowledge on this subject. Those who do not know about sex or make fun of it, are ignorant, stupid, small-minded”, says Dr. Shereen El Feki. “For our ancestors, sex was a source of happiness, creativity and vitality. Now it is a taboo, and it is problematised in the Arab media”. The mediaeval Arab writer Umar Muhammad al-Nafzawi, who authored the sex manual The Perfumed Garden, would probably turn in his grave at the sexual mores of today’s Arabs.

El Feki, a Canadian-Egyptian immunologist (University of Cambridge) and award-winning journalist for The Economist and Al Jazeera, spent the past five years taking the temperature in bedrooms across the Arab world - a region spanning 22 countries and numbering 350 million people, in which the only acceptable, socially acknowledged context for sex is marriage... state-registered, family-approved, religiously sanctioned marriage. Anything else is *ayb* (‘shameful’), *illit adab* (‘impolite’) or *haram* (‘forbidden’).

The result of El Feki’s quest is a must-read, a One Thousand and One Nights of hard data, polls and intimate testimonies, well-researched journalism and a personal memoir. Sex in the Arab world is an enigma, an Egyptian gynaecologist explains to El Feki: “It is the opposite of football. Everyone talks about football, but hardly anyone plays it. But sex - everyone is doing it, but nobody wants to talk about it”. In spite of this habitual reticence, El Feki was able to explore the substance of contemporary sex life in the Arab world, from Tunisia over Egypt and Saudi Arabia to Qatar.

Across that vast region, the sexual experience is shifting, albeit at a tectonically slow pace. Sexual freedom still defines the West, as the Orient seems stuck in a state of sexual lockdown. Not that long ago, the perception was inverses. In the eyes of the 19th-century West, the Arab world conjured up highly eroticised visions of mystery and loose morals, sensuality and sex.

“The reality today in the Arab world is far removed from that vision”, says El Feki. “As a Muslim who grew up in Canada, I prefer to think of religion and intimacy as private matters. My book argues for a change along those lines, but within an Islamic context. It would be utter nonsense to argue for a secular sexual revolution in the Arab world. My book does not seek to stigmatise today’s Arabs, but instead hands them some talking points, as a start to help change the region’s sexual politics. For this reason, the book has a companion website (www.sexandthecitadel.com), which presents a lot of additional facts and figures on the topics at hand”.

El Feki grew up in Canada, the daughter of an Egyptian father and a Welsh mother. Her Muslim roots fed her interest in the Arab world. “After 9/11, I wanted to understand my Arab heritage better. First, I went to work for Al Jazeera as a presenter. In 2007, I started researching *Sex and the Citadel*. I’ve combined that research with advocacy on HIV/AIDS, serving as vice-chair of the UN's Global Commission on HIV and the law (www.hivlawcommission.org), which advocates legal reform around the world, including of laws regulating sexuality. This gave me access to information that is hard to come by in the Arab world, because sex research is scarce. Many pressing questions have not been addressed yet, and results often end up locked away in a desk somewhere”.

## One of these pressing questions concerns the rate of infertility.

“Officially it is on a par with the global average - one in eight couples is infertile. But the statistics are not really reliable. How often is infertility actually diagnosed? We do not know. Most women do not talk about sex to their gynaecologist, and their doctors are not really open-minded about the subject”.

“Researching sex in the Arab world is far from easy. It is hard to get any insight. Not only because of the taboos around sex, also because there is no culture of publishing or sharing info. There are hardly any journals writing about sex. Governments sit on the results of the surveys they ordered. There is no culture of transparency. The biggest pool of data about sex comes through HIV research. It was absolutely not easy to get hold of that information.”

**Figure:**
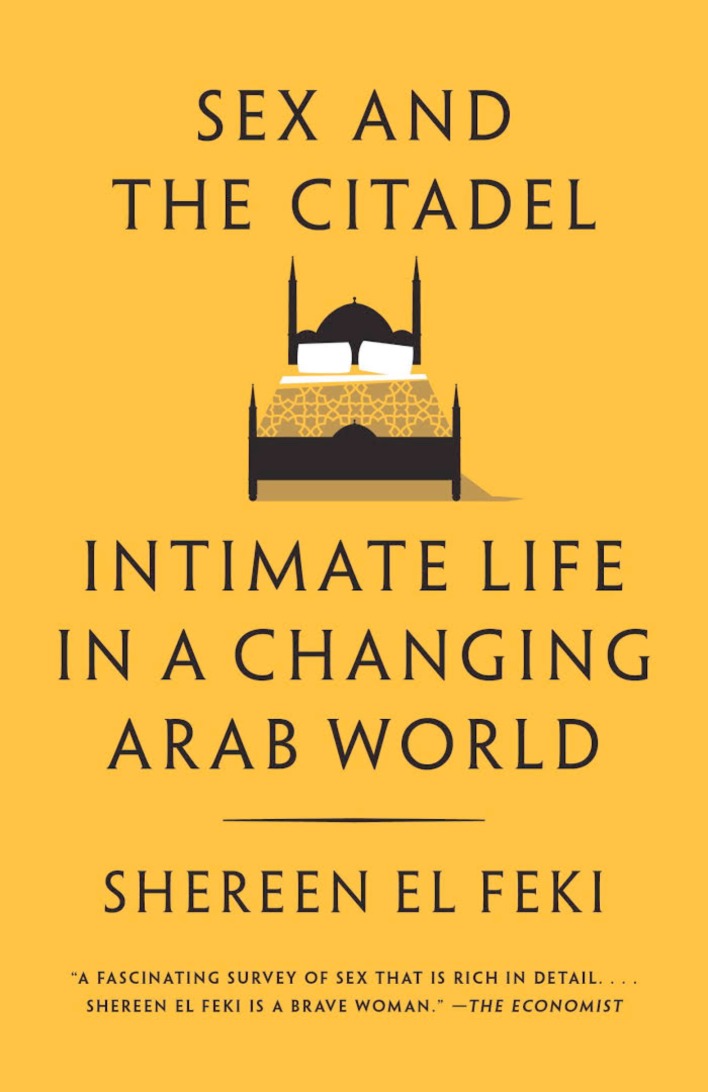


## In most of the Arab world, infertility is a woman’s problem.

“Being able to procreate is so crucial to men’s self-image that they find it hard to accept anything less. Yet male infertility is rampant. Dr. Amira Badr al-Din Mehany, who heads the embryo lab at the assisted reproduction unit of Al-Azhar University (www.alazhar-iicpsr.org/assisted), told me how amazed she had been at the rate of male infertility when she started eight years ago”.

## What are the causes?

“Nobody knows for sure. Dr. Mehany told me smoking and pollution are likely causes. Others think the cause may be genetic, due to the high rate of consanguineous marriages, which increase the likelihood of genetic defects being passed on to children. Other possible causes mooted are wearing jeans or exposure to agricultural chemicals. The latter would explain the large number of small farmers turning up at Dr. Mehany’s department. While there are several private IVF clinics in Egypt, a single treatment cycle there would cost more than €1.000 - a small fortune. At Al-Azhar, the treatment is a third of that price, and the poor receive further discounts”.

## Do infertility treatments in Arab countries face any other challenges?

“Like other bodily fluids, semen is considered ritually impure in Islam. So men and women have to bathe after intercourse. Women shower almost immediately after sex. Infertility specialists advise to wait at least half an hour after ejaculation, which many women find disconcerting. Even more problematic is getting a sperm sample. Many men consider masturbation deeply troubling, as they blame their infertility on it. Most religious scholars consider it *haram*. So many men have a problem with producing a semen sample, even for infertility treatment. Often, they fail to produce a sample at all”.

## Are there religious limits to assisted reproduction?

“Very much so. In Egypt, sperm or egg donation and surrogacy are unacceptable because it can lead to an illegitimate child. Techniques that require a couple’s own gametes - in vitro fertilisation, artificial insemination and ICSI - are okay, but are restricted to married couples”.

## Did your expectations of what you would find in the Arab bedroom match your ndings?

“Having grown up with one foot in the culture, I knew there were a lot of taboos. What I had not expected, were the many individuals in Morocco, Tunisia, Algeria and other countries trying to push the boundaries, in so many directions. The creativity and innovation in matters of sex education were remarkable. The glass is not always half empty, I discovered. Sometimes, it is half full...”

“That is what many in the West do not seem to notice. They focus on the problems in the Arab world while many in the Arab world are focusing on the solutions. It is obvious that there are problems, but one of the messages of my book is that the solutions people are finding are okay. The Arabs do know what they are doing. They just do things differently”.

## One reviewer called your book a depressing read.

“Quite the opposite, I think. But I do not want to knock people over the head. That is why I did not compare the sexual practices in the Arab world with those in the West or in other parts of the world. Many of my readers will live outside the West, so why always compare to Europe or North America?”

## Many people will be happy to see that the Arab world has not always been locked in these patterns of denial and suppression.

“The background I bring is very useful. And I am not suggesting we go back to a mythical golden age of sexual liberation in our past. It did not exist. But there was more openness. And you do not have to look back to the period of the Abbasid caliphate, but to our own fathers’ and grandfathers’ time. My own grandmother had a distinct lack of embarrassment about these issues, while many women today are bottled up and conflicted about sex”.

## The American thinker Howard Bloom blames that repressed attitude on the lack of hugging in the Arab World.

“I never heard that before. But indeed, physical affection in public gets rarer. Ayman Zohry, an expert on Egyptian migration, told me a remarkable story. He comes from a village where a large proportion of the men migrate to the Gulf for work. Twenty years ago his female relatives hugged him when he returned to the village. Now they do not. Many women will not even shake hands with a man. The Palestinian Safa Tamish, who runs Muntada Jensaneya, the Arab Forum for Sexuality, Education and Health (www.jensaneya.org), has a sexual education programme. She found out that husbands and wives find it easier hugging each other in front of the kids. And that those displays of affection utterly changed the dynamics in the family. You could feel the love and companionship and everything in the family changed. Changes in the intimate life led to changes in all aspects of life”.

**Figure:**
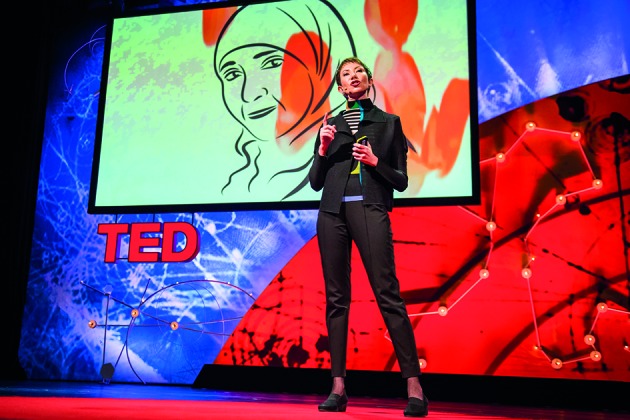


## Has hugging in Tahir Square changed life in Egypt?

“You can see many more couples holding hands and hugging in public now. This never happened before the uprising, not in broad daylight anyway. You see these small changes everywhere. In Morocco, there was a Kiss-In. There is a lot of tension between the public and the private, but people are starting to question the old taboos. But in a very subtle way, it is not a full-frontal assault on sexual morality”.

## Femen is a non-starter, then.

“Exactly. It is so alien to the way we see changes in the Arab region. It is actually quite damaging. In the West, there is a more confrontational approach to change, but not so in the Arab world. It takes very gradual steps. Certainly in sexual life, which is bound up in politics, economy and religion”.

## Does the rising rate of HIV infections reflect the change in sexual conduct?

“I think so. According to the latest UNAIDS report, published this September (www.unaids.org), there are only two regions in the world where HIV infection is on the rise. One is the Arab world. If you look at the curves of the graphs, you see them shooting up. In Tunisia, 15% of the men are infected. There is such a stigma around HIV and AIDS in the Arab world that people are treated too late. And the epidemic is already being feminised”.

## How do you mean?

“The classic story is that of a woman who is married and in her early twenties. Because of the pressure to have a child, she gets pregnant soon. The child is unwell, and in hospital they discover mother and baby are HIV-infected. And the husband too. For the woman, this is a bolt from the blue. She has only had sex with her husband. In many cases, however, the doctors will tell the man he is infected, but not the woman”.

## Due to the stigma attached to HIV?

“An infected woman is generally considered to be a ‘bad’ woman. She must have engaged in extramarital sex. The level of tolerance for women is extraordinarily low. The same goes for drug users, a growing problem in the Arab world, particularly in Egypt and Libya. For women, it is socially unacceptable. So men will be sent to a rehabilitation centre, but not women. HIV and drug abuse go hand in hand. Condoms are never used, so the infection spreads easily”.

## Can HIV be stopped in its tracks?

“Yes, of course - even if there is a lot of unprotected sex amongst young people. And despite the toxic mess caused by the lack of proper education, the taboo around contraceptives and the illegal status of abortion. The tragedy is that it will require money, focus and political will, all of which are in short supply. HIV is the measure of all your other problems, a mirror to a society. Morocco and Oman have stepped up to the plate, Tunisia and Algeria have a solid track record. But Egypt is problematic because it does not qualify for many international funds and bilateral aid”.

## Which country has made the greatest progress?

“To everyone’s surprise: Saudi Arabia. They have a unusually talented woman in charge of their AIDS programme”.

## But again: hard data about HIV are the problem?

“Yes. As a journalist, I discovered a massive gap between official statistics and private reality. While people were assuring me that HIV was not a problem in the Arab world, I met entire families who were infected. This is what set me off to write the book - the realisation that sex is the wedge between appearance and reality in Arab societies. There is a collective unwillingness to face up to any behaviour that falls short of the marital ideal. As a consequence, all data on sex coming out of the Arab world have to be taken with a grain of salt”.

## You describe the difference between the various Arab countries concerning the attitude towards sex.

“They cannot really be described on a spectrum from strict to loose. There is a lot of variation inside each country. And we lack robust empirical research. My book has some, but it is largely anecdotal. There is no ranking of how sexually messed-up Arab countries are (laughs). We do not know the level of sexual angst or confusion. But we have insights into sexual violence. About a third of women have experienced domestic violence within the last year. About 10 to 20% say they have experienced sexual violence. But quite possibly, these numbers are underreported”.

**Figure:**
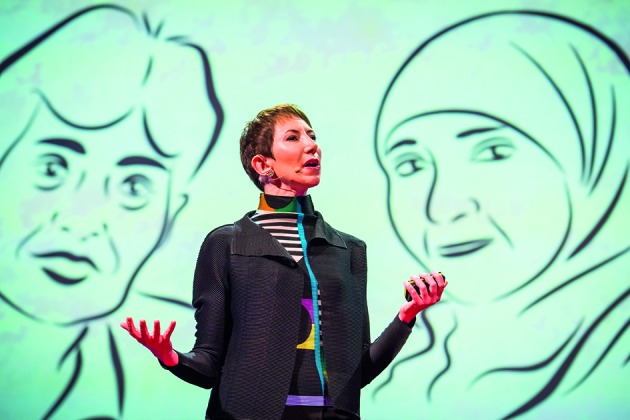


## So there are no reliable data on the level of sexual practice?

“Not really. We have some information on attitudes, though. When you compare the ability to mobilise social groups, we can say that the relatively more open societies are Morocco, Tunisia and Algeria. Jordan is quite open on some issues like honour killing, which is a real problem there. Lebanon is also relatively open; the Lebanese student population must be the most studied segment of Arab society as a whole when it comes to sexual attitudes and behaviour”.

## What do the latest studies tell us?

“They deal with virginity and the acceptability of premarital sex. No less than 70% of men said it was acceptable, against less than 30% of women. But the majority of men said they wanted to marry a virgin. So there you go. These studies should be taken with a grain of salt; their results depend heavily on who asks the questions, and what the respondents think they want to hear”.

## How do we figure out what is actually going on, then?

“You can get a sense by looking beneath the surface. Kuwait is considering a law that would allow gender testing. In the book, I talk about their punishment for cross-dressers. The question is: What is going on? Why this fixation on transgender people, at the same time as the Arab world is in such political upheaval?”

## The Arab world is rife with sexual stereotypes.

“Definitely. They say that Moroccan women are a little light on sexual morals. As a leading Moroccan magazine put it: To be Moroccan is not a nationality, but a conviction. That about sums it up. Moroccan women are politically more liberated, so they must be sexually liberated too. That idea is wrong, of course. Many people who are currently fighting for political liberalisation are absolutely horrified by the idea of sexual freedom. Tunisian women have a reputation similar to that of Moroccan women. And you get the impression that men from the Gulf area must all be gay (laughs)”.

## Who are these people who are pushing the boundaries?

“There are a lot of them. Aliaa Elmahdy is one example. She is the ‘Nude Revolutionary’, who achieved international notoriety when she posted photos online of herself naked. Frankly, I do not think this is how you achieve change. Another example is the Moroccan Kiss-In. On Facebook, thousands of people said they would attend and participate. But what happened? Only 12 people showed up. This slacktivism is typical. Most Arabs are willing to push the boundaries online, but not in real life”.

## Double standards seem to be the norm in the Arab world.

“There is one set of ethical standards for men - and a completely different one for women. There is no equality of expectations, and what happens in private does not match up with what is expected in the public sphere. Take, for example, zawai misyaf, the so-called ‘temporary marriage’. Basically, it is prostitution, people trafficking and sex tourism masquerading as marriage. But as long as it looks like a ‘proper’ marriage, nobody questions it. These marriages can last a few days, a couple of weeks at most”.

## Who are these temporary husbands and wives?

“In Egypt, I spoke to a pimp. Before the revolution, he organised at least a thousand marriages a year. Some were to wealthy Saudi men, who like to have a teenage girl, or even more specifically a virgin, and then keep her for, say, 10 days to two weeks. He knew the parents who sell their daughters for this kind of arrangement. A contract is made up, and the girl is brought to her ‘husband’. Some girls have up to half a dozen ‘marriages’ a year”.

## You talked to Samia, one such ‘bride’.

“She had had three summer marriages over the course of two years. She had left school at 12. Research shows that a majority of these holiday matches involve girls younger than 16. In Samia’s village, 70% of the girls had temporary marriages, she said. Violence, unprotected sex and sexually transmitted diseases are standard ingredients of their stories. Samia herself was ‘sick in sex’ after one of her marriages. Nevertheless, she hoped for marriage - after an operation to restore her hymen”.

## An example of the absurd lengths to which the Arab obsession with virginity can go.

“Virginity is defined as an anatomical state, not as a habit of chastity. This has devastating consequences, whether in the shape of female genital mutilation, virginity testing or other unsafe practices young people are engaging in, like unprotected oral and anal sex, or superficial relationships”.

## So how do you reconcile the reality of the bedroom with outward appearance?

“First, one has to avoid being accused of selling out to the West. This is a highly emotive issue. We should not only turn to the past for solutions, but Muslim culture since the beginning did have some healthy approaches to sex. We know some things are *halal* and others are *haram*, but in between, there are 50 shades of grey, and more. At times, people have gravitated towards a pragmatic approach towards sex. Now, we’ve moved towards a stricter approach. Why does this happen? Do we seriously need to? Are there alternatives? I, for one, think so. Take abortion, for example. In most Arab countries, it is illegal. But in Algeria, it is not. This shows there is a wide range of interpretations possible within Islamic jurisprudence”.

## So you are an optimist.

“Because there’s reason for optimism. The Arab world is not a hopeless place. Gradually, we’re laying the foundation for a public discussion of sex. That change will not be a revolution. It will take decades. Many people in the West think this impossible, but they forget that their own sexual revolution was no helicopter that simply lifted off from taboo land and flew to the realm of sexual freedom. In the West as well, the sexual revolution was a giant Hercules transport plane that was able to take off thanks to a long runway. That runway represents hundreds of years of political, economic and social changes.

## If you could change only one thing, what would it be?

“The way Muslim women educate their sons. That definitely has to change. They should raise them without privileges, teach them to treat their sisters equally and face up to their responsibilities. If that would happen, the rest would follow. But this process would also take time, and effort: the patriarchal structure also affords certain advantages and privileges to many women...”

